# Cost-effectiveness of a nurse-based intervention (AIMS) to improve adherence among HIV-infected patients: design of a multi-centre randomised controlled trial

**DOI:** 10.1186/1472-6963-13-274

**Published:** 2013-07-17

**Authors:** Edwin Oberjé, Marijn de Bruin, Silvia Evers, Wolfgang Viechtbauer, Hans-Erik Nobel, Herman Schaalma, Jim McCambridge, Luuk Gras, Eric Tousset, Jan Prins

**Affiliations:** 1 Department of Communication, University of Amsterdam, Amsterdam School of Communication Research ASCoR, Kloveniersburgwal 48, 1012 CX, Amsterdam, Netherlands; 2 Department of Health Services Research, Caphri, Research School on Public Health and Primary Care, Maastricht University, Maastricht, Netherlands; 3 Department of Psychiatry and Psychology, Maastricht University, School for Mental Health and Neuroscience, Maastricht, Netherlands; 4 Department of Internal Medicine, Division of Infectious Diseases, Center for Infection and Immunity Amsterdam, Academic Medical Center, Amsterdam, Netherlands; 5 Department of Work and Social Psychology, Maastricht University, Maastricht, Netherlands; 6 Faculty of Public Health and Policy, London School of Hygiene and Tropical Medicine, London, UK; 7 Stichting HIV Monitoring, Amsterdam, Netherlands; 8 AARDEX Group Ltd., a MWV Healthcare Company, Visé, Belgium

**Keywords:** Adherence, AIMS, Economic evaluation, HIV, Intervention, Randomised controlled trial

## Abstract

**Background:**

Non-adherence to HIV-treatment can have a negative impact on patients’ treatment success rates, quality of life, infectiousness, and life expectancy. Few adherence interventions have shown positive effects on adherence and/or virologic outcomes. The theory- and evidence-based Adherence Improving self-Management Strategy (AIMS) is an intervention that has been demonstrated to improve adherence and viral suppression rates in a randomised controlled trial. However, evidence of its cost-effectiveness is lacking. Following a recent review suggesting that cost-effectiveness evaluations of adherence interventions for chronic diseases are rare, and that the methodology of such evaluations is poorly described in the literature, this manuscript presents the study protocol for a multi-centre trial evaluating the effectiveness and cost-effectiveness of AIMS among a heterogeneous sample of patients.

**Methods/design:**

The study uses a multi-centre randomised controlled trial design to compare the AIMS intervention to usual care from a societal perspective. Embedded in this RCT is a trial-based and model-based economic evaluation. A planned number of 230 HIV-infected patients are randomised to receive either AIMS or usual care. The relevant outcomes include changes in adherence, plasma viral load, quality of life, and societal costs. The time horizon for the trial-based economic evaluation is 12–15 months. Costs and effects are extrapolated to a lifetime horizon for the model-based economic evaluation.

**Discussion:**

The present multicentre RCT is designed to provide sound methodological evidence regarding the effectiveness and cost-effectiveness of a nurse-based counselling intervention (AIMS) to support treatment adherence among a large and heterogeneous sample of HIV-infected patients in the Netherlands. The objective of the current paper is to describe the trial protocol in sufficient detail to allow full evaluation of the quality of the study design. It is anticipated that, if proven cost-effective, AIMS can contribute to improved evidence-based counselling guidelines for HIV-nurses and other health care professionals.

**Trial registration:**

The study has been registered on clinicaltrials.gov (Identifier: NCT01429142).

## Background

In patients infected with HIV, viral replication can be effectively suppressed with antiretroviral therapy (ART), allowing the body’s immune system to restore and function adequately [1]. Other primary outcomes of HIV treatment are improved patient health, life expectancy, and quality of life (QoL) [2]. Moreover, HIV transmission risks are reduced substantially in patients with lower viral loads, which is beneficial from a public health perspective [3-5]. Although full viral suppression can be achieved by (some) patients with lower adherence levels (70-90%) [6-8], it is generally recommended that patients take at least 90-95% of their medication for long-term suppression of viral replication, to minimise the risk of developing viral resistance [9-11]. Meta-analyses have shown that the quality of adherence support delivered by health care professionals during usual care is key to achieving adequate adherence levels and viral suppression [12,13]. Hence, in addition to the advances in the available ARVs, high-quality adherence support is a cornerstone of the major advances made in improving the lives of people living with HIV, and slowing down the spread of (resistant) HIV.

Non-adherence is associated with lower quality of life, morbidity, mortality, avoidable productivity losses, and health care costs [14-17]. In spite of the fact that many HIV-clinics may currently offer high-quality ‘usual adherence care’, achieving and/or sustaining high levels of adherence remains a challenge to a substantial number of patients [12]. Hence, specially developed adherence interventions can still make a relevant contribution, even in settings that already offer high-quality usual adherence care [18].

Numerous HIV-treatment adherence interventions have been developed and evaluated [12,13,19-24]. For interventions to be assessed as effective, they have to be superior to the usual care already provided in the clinic where the intervention is studied [12,13]. Intervention programmes also need to be well-accepted by patients and clinicians, and tailored to the capacity of the clinics delivering HIV-care in order to be sustainable [25]. Ideally, interventions should also provide proof of cost-effectiveness among a representative sample of patients and clinics before they are implemented in routine practices [26,27].

A recent systematic review of cost-effectiveness evaluations of adherence interventions among patients with other chronic conditions included thirteen randomised controlled trials (unpublished data). Only one trial evaluated cost-effectiveness of an adherence intervention among patients treated for HIV [28]. The objective of this review was to critically assess the trials with the Cochrane risk of bias tool and the Drummond checklist for the quality of economic evaluations, and examine cost-effectiveness outcomes [29,30]. Risk of bias varied considerably between trials. Moreover, a key issue was the lack of (accurate) reporting of the relevant research methodology: for example, in 24% of the cases the Cochrane bias score could not be directly coded (‘Unknown’). Regarding the quality of cost-effectiveness evaluations, an average of 44% of required elements for a sound economic evaluation were reported. However, it was often not clear from the articles whether elements were merely not reported or not performed. The authors therefore concluded that, amongst others, cost-effectiveness evaluations of future adherence trials should be more comprehensive, and that trial reports should improve to allow more detailed evaluations of their methodology. Publishing the full study protocols that specify the objectives, study design, primary outcome measures, and analysis strategy is therefore key. The objective of the current paper is to describe a trial protocol of a cost-effectiveness evaluation of an HIV-adherence intervention that reports the information required to fully evaluate the study design.

### The adherence improving self-management strategy (AIMS)

One of the few adherence interventions that showed positive effects on both adherence and clinical outcomes (in this case, undetectable HIV loads) is the Adherence Improving self-Management Strategy (AIMS) [18,31]. This intervention has been designed by behavioural scientists and clinical staff, and was then adapted based on patient feedback [18,31]. Central to AIMS is electronic adherence monitoring with MEMS-caps (Aardex Ltd.). MEMS-caps are electronic pill caps that register date and time of pill bottle opening. The data obtained via the MEMS-caps can be printed in simple but comprehensive plots that show medication intake patterns over long time-periods, allowing for a detailed analysis of adherence barriers and potential solutions to deal with these for each individual patient. Discussion of MEMS-data has been combined with HIV-nurses’ usual adherence support practices, and enhanced with motivational and self-management techniques [31]. During a pilot-study and randomised controlled trial, both health care professionals and patients evaluated AIMS positively. Moreover, AIMS has been shown to enhance adherence and viral suppression rates among 133 patients in a 9-months randomised controlled trial [18]. Upon request of the health care professionals, who wanted to first master the counselling intervention before applying it in - for them - more challenging populations or circumstances, the trial was set up in two stages. In the first stage, only Caucasian patients were included, and patients with untreated drug addictions or untreated severe mental illness were not approached. In the second stage, these restrictions would be removed. Unfortunately, slower than anticipated inclusion rates, changes in the nursing team, and lack of budget prevented progression to stage two. In addition, the trial did not include a cost-effectiveness evaluation. For these reasons we decided to seek additional funding to conduct an economic evaluation of the AIMS intervention among a heterogeneous sample of patients. The present manuscript describes the study protocol and the rationale behind the design choices.

## Methods/design

The study was primarily designed by the core team of the current project, consisting of three behavioural scientists (authors MdB, JM, and HS), an infectiologist (author JP), a nurse practitioner (author HEN), a health economist (author SE), and three statisticians (authors WV, LG, and ET). The team made decisions on the study design and outcome measures regarding the effect- and economic evaluations. After recruitment of seven HIV-clinics for collaboration in the present multi-centre clinical trial, infectiologists and HIV-nurses from these clinics were visited twice to provide them with details regarding the proposed study design. During these meetings, members from the collaborating HIV-clinics were invited to provide feedback on inclusion criteria and study procedures. This input resulted in various improvements in the project design and was expected to result in more commitment to the project from the participating clinics.

The study has been approved by the ethics committees of the Academic Medical Center Amsterdam, the Netherlands, and of each participating centre, and registered on clinicaltrials.gov (Identifier: NCT01429142) prior to study start.

### Research questions

The overarching research questions addressed by this project are:

*Effectiveness evaluation*

1. What are the effects of the AIMS-intervention in comparison with usual care with respect to changes in plasma viral load (primary outcome) and adherence (secondary outcome)?

*Economic evaluation*

2. From a societal perspective, is the AIMS-intervention, in comparison with usual care, preferable in terms of costs, effects, and utilities? (trial-based economic evaluation)

3. What is the incremental cost-effectiveness ratio (ICER) of the AIMS-intervention in comparison with usual care? (trial-based economic evaluation)

4. In comparison with usual care, is the AIMS-intervention preferable in terms of costs and utilities during the remaining life expectancy of the study population? (model-based economic evaluation).

### Design

**Figure 1:**
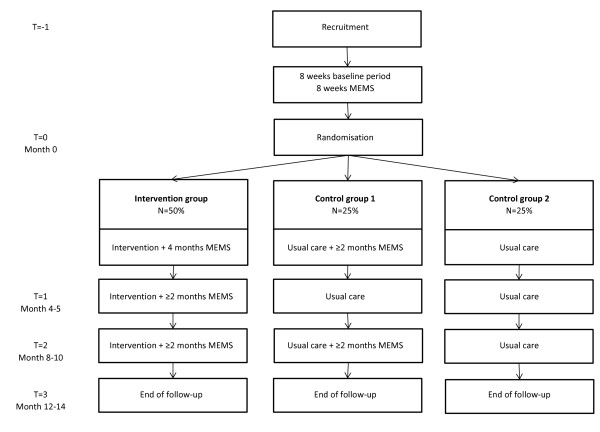
Trial flow for treatment-experienced patients.

**Figure 2:**
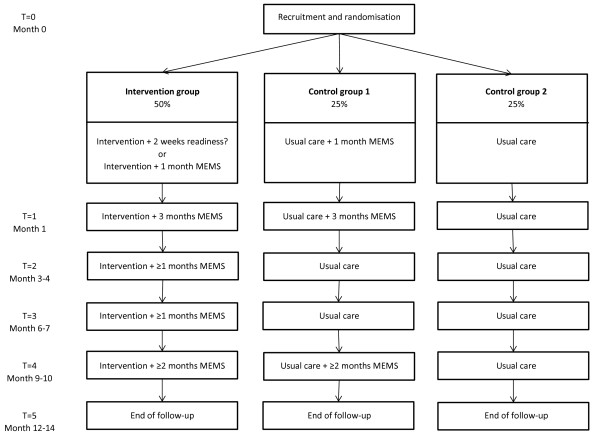
Trial flow for treatment-initiating patients.

The study uses a multi-centre RCT design. Participants are randomised to either the AIMS-intervention or to usual care. The trial flow and randomisation procedures are shown graphically in Figure 1 (for treatment-experienced patients) and in Figure 2 (for treatment-initiating patients). Embedded in this RCT is a trial-based economic evaluation and a modelling study to investigate the cost-effectiveness and cost-utility of the AIMS-intervention versus usual care.

### Participants selection and inclusion criteria

The study focus was initially on treatment-experienced patients (as was the case in the previous studies), but after visiting the participating clinics for their input on the study protocol, the decision was made to also include treatment-initiating patients. The major arguments for facilitating this change was that [a] it increases representativeness of the study sample, and [b] substantial gains can be made in treatment-effectiveness in the first year of the treatment by enhancing involvement in care and adherence to treatment (i.e., about 75% of the patients who started medication achieve the treatment objective of a plasma viral load concentration of 50 copies/ml or less within 9 months; Monitoring Report 2012 p68, http://www.hiv-monitoring.nl/index.php/nederlands/.

Given that the majority of the treatment-experienced patients adhere very well to the treatment and have a suppressed viral load [18], two selection criteria were developed to select those patients who are more likely to benefit from the intervention. A first selection was that only those patients who had a detectable viral load in the past 3 years (with the count starting after the first 9 months of treatment to allow them to achieve an undetectable viral load) are asked to participate in a baseline adherence monitoring stage. After a minimum of 2 months baseline adherence monitoring period (long enough for any measurement effect on adherence to dissipate) [18,32], only the patients with suboptimal adherence during baseline are randomised. The data used to determine these cut-off points come from the Stichting HIV Monitoring (detectable viral load in the previous 3 years) and the prior RCT (suboptimal adherence) [18].

Stichting HIV Monitoring collects anonymous routine clinical data from all HIV patients in clinical care in the Netherlands. Using this database, the probability of a viral load measurement of 50 copies or more during one year of follow-up was estimated as a function of the time since patients’ last detectable viral load prior to the one year follow-up period (N = 4440). The analyses showed that the more recent patients had a detectable viral load ≥50 copies/ml prior to the 12-month follow-up, the larger the probability that they would have a detectable viral load during the 12 month follow-up (unpublished data). Based on these analyses, we decided that a meaningful cut-off was to include patients who had a detectable viral load ≥50 copies/ml in the past 3 years. Regarding adherence, secondary analysis of the baseline data from the prior RCT were used to identify the most meaningful cut-off. These analyses revealed that for a BID regimen a cut-off of 95% adherence predicted the next viral load (detectable-undetectable) best, and for a QD regimen this was 99%. The difference between QD and BID can be explained based on the duration of drug action versus the probability of patients skipping one or multiple doses sequentially [33]. Hence, treatment experienced patients with a detectable viral load during the last 3 years (after the first 9 months of treatment) were selected initially, and from those patients with suboptimal adherence (≤ 95% for BID or < 100% for QD) during the baseline assessment are included and randomised.

Besides the inclusion criteria as described above for treatment-experienced patients, treatment-initiating patients were eligible for participation without any inclusion criteria. The exclusion criteria for both treatment-experienced and treatment-initiating patients are: (1) age <18 years, (2) severe psychiatric disorders precluding compliance with study procedures, (3) pregnancy, (4) plan to interrupt treatment or change clinics in the next fourteen months, (5) life expectancy less than one year as determined by physician, (6) not able to communicate with health care professionals without an interpreter, (7) not taking medication autonomously.

### Recruitment

Participants are recruited from seven HIV-clinics in the Netherlands: The Academic Medical Center Amsterdam, the Leiden University Medical Center Leiden, the Erasmus Medical Center Rotterdam, the Slotervaart hospital Amsterdam, the St. Lucas-Andreas hospital Amsterdam, the Isala clinics Zwolle, and the HAGA hospital The Hague. The first three are academic hospitals and the other are non-academic hospitals. In total these centres have approximately 5500 patients in care and each year about 350–400 patients initiate treatment.

Estimates of recruitment rates were based on Stichting HIV Monitoring data and the prior RCT [18]. About 50-60% (i.e., 3000) of the 5500 treatment-experienced patients in the eligible clinic were estimated to either have a detectable viral load in the last 3 years or to have a detectable viral load during the study inclusion period. Assuming a 50% refusal rate and 50% of the patients to have excellent adherence [18], this leaves a sample of 1500/2 = 750 potential treatment-experienced study participants for randomisation. The planned recruitment period was 16 months, based on the recruitment rates in the prior RCT. Due to various external influences during the start-up of the project (e.g., a large clinic had a personnel shortage and started a year later), the inclusion period was extended to 22 months.

The HIV-nurses in the clinics manage recruitment. During routine clinical visits, they approach eligible patients and inform them verbally about the content and objectives of the project. These objectives were formulated in terms of ‘improving the quality of care’ rather than ‘improving adherence’, in an attempt to cover up the specific study hypotheses. Patients are also given the information in writing and the standard 2-week period for deciding on study participation according to the Dutch ethical guidelines. Patients are at liberty to decide to start immediately, knowing they can withdraw at any time without any consequences for their treatment.

### Sample size

During the initial grant application, a sample size was computed based on the assumption that only non-adherent, treatment experienced patients would be approached. However, following the input from participating clinics, the final inclusion and exclusion criteria were formulated as explained above. An updated sample size computation was done after inclusion had been initiated, which was submitted to (date: 27^th^ February, 2013) and accepted by (date: 15^th^ April, 2013) the funder (ZonMw, the Netherlands Organisation for Health Research and Development) using actual trial data in order to enhance accuracy of the estimates (the expected treatment effect is the same in both computations, the other estimates were adjusted to match then new criteria and available data). The trial summary on clinicaltrials.gov includes both the initial and later the updated computations.

The updated sample size computation is the following: A sample of 230 patients (22 nurses) is required to obtain 80% power to detect a significant intervention effect on viral load for at least one of three time points (for treatment-experienced patients: T1, T2, and T3; for treatment-initiating patients: T3, T4, and T5) with alpha = .05 (two-sided) and using a Bonferroni correction. Viral load at baseline is used as a covariate. A multilevel model is used with random intercepts and random treatment effects at the nurse level. The sample size calculation is based on the following assumptions: depending on the nurse, (a) 60% to 80% of treatment-initiating patients have an undetectable viral load during usual care (i.e. ≥ 6 months after start of ART), (b) 15% to 20% of treatment experienced-patients and all treatment-initiating patients have a detectable viral load at baseline, (c) this increases in the intervention condition compared to the control condition by 5 to 20 percentage points, (d) a nurse recruits on average 11 patients for the trial, and (e) an expected maximum dropout of 10%.

### Randomisation procedure

Discussions with clinicians revealed that it is not desirable to randomise clinics (out of fear for recruitment bias) or nurses (logistically challenging, i.e. what to do when an intervention patient comes in and their nurse is ill, and only a control group nurse is present?). Hence, the decision was made to individually randomise patients within nurses, so that each nurse sees both intervention and control participants.

Experienced patients with insufficient adherence during the baseline measurement and treatment-initiating patients are randomised 1:1 to either the intervention or control condition. For each HIV-nurse, a random allocation scheme, stratified for treatment-experienced and treatment-initiating patients, is generated using computer-generated blocks to balance treatment and control assignments within nurses. The size of the blocks is random (block sizes of four, six, and eight; the clinicians are not made aware of this). Because of the combination of stratification, randomly-ordered block sizes, and the control-group blinding procedure described in the next paragraph, it seems impossible for clinicians to predict consecutive treatment assignments. In practice, the randomisation is fully automated using the web-platform medAmigo™ (Aardex Group Ltd.).

Two additional procedures are integrated within the randomisation process. First, the study is powered based on the primary outcome measure of viral load. Data from the prior RCT suggests that in order to evaluate the effects on adherence a smaller sample size suffices. In order to reduce the study burden for both patients and clinics, it was therefore decided to monitor adherence among only half of the control group (since MEMS-monitoring is part of the intervention, all intervention participants were monitored). Hence, control participants are assigned randomly to using the MEMS-cap or not using the MEMS-cap (control groups 1 and 2: see Figures 1 and 2). Second, 33% of the treatment-experienced patients with adequate adherence at baseline (>95% for BID and 100% for QD) are being randomised to the control groups in order to blind clinicians to the adherence status of control participants (since non-adherence is an inclusion criterion). Naturally, the treatment-experienced patients with adequate adherence at baseline are not taken into account for effectiveness and economic evaluation.

### Intervention and usual care

Although the overall intervention strategy is similar for treatment-initiating patients and treatment-experienced patients, the first intervention visit is different for both groups. Below, presented first is the description of the intervention as provided to treatment-experienced patients. Subsequently, the elements are described that are unique for treatment-initiating patients.

#### The AIMS-intervention

The AIMS-intervention is a nurse-delivered, adherence-supporting intervention, with MEMS-data printed in simple but detailed plots to assist the counselling strategy. For the rationale behind the intervention, please see de Bruin et al., 2005; 2010 [18,31].

The intervention protocol below describes the main steps taken with those patients randomised to the intervention group after their initial two months of MEMS-baseline monitoring. The intervention is available on-line through the web-platform medAmigo™ and can only be accessed by downloading the MEMS monitors for patients assigned to the intervention arm. The software used for randomisation is also the software that guides the HIV-nurses and patients through the intervention. Each of the following intervention steps is shown on a separate webpage:

1. The HIV nurse discusses practical, tailored information regarding adherence and the consequences of (non)adherence (verbally and using a simple graph).

2. The HIV nurse asks the patient to define a desired level of adherence, based on seven exemplar MEMS-reports varying from ‘regular and no missing doses’ to ‘irregular and many missing doses’.

3. If a patient selects an insufficient level of adherence (e.g., several missing doses; in practice this occurs rarely), the HIV-nurse explores the reasons for this with the aim to motivate the patient to strife for a safe level of adherence.

4. After a patient selects the desired level of adherence, the nurse enquires about the patients’ personal drives and motives (e.g., ‘Why this level and not lower?’).

5. The patients’ own MEMS-data (i.e., actual adherence level) is printed, and discrepancies between the desired (step 4) and actual adherence is discussed, appealing to the patients’ responses to Step 4 when a lack of motivation seems to be an underlying issue.

6. The HIV-nurse and patient use the MEMS-report to identify patterns of non-adherence (e.g., weekends or regularly returning occasions) and the cause of this pattern; or the reasons for the most recent occurrences of non-adherence if there is no obvious pattern. The patient is encouraged to identify solutions to deal with these problems, and the software offers successful strategies from other patients.

7. The nurse and patient finish this session with developing an action plan for the next period, including an overall behavioural objective (e.g., no missed doses) and detailed actions for how to achieve this. If the behaviour requires the development of a routine, if-then plans are formulated [34]. The patient is offered a MEMS-view cap, which has a display on top showing how often the bottle has been opened that day. This serves as a direct feedback mechanism for missed doses, supporting the self-monitoring of adherence.

8. During subsequent intervention sessions, nurse and patient evaluate whether the action plan was successful, whether the patient has encountered difficult situations and how they were dealt with, followed by printing and discussing the new MEMS-report. If adherence problems remain, potential causes and solutions are discussed and formulated in action plans until the patient reaches the desired adherence level.

9. When a patient has successfully improved adherence, goals are set for the maintenance of behaviour and to develop strategies for risk situations that may disrupt the new routine.

The aim in this study is that patients reach their desired level of adherence during the first 4–5 months of the intervention, strive for behavioural maintenance (i.e., make the achieved level a routine) during the next 4–5 months, followed by a follow-up period of another 4–5 months.

### Adapted elements for treatment-initiating patients randomised to the intervention

Generally, two trajectories can be distinguished with respect to ART initiation. First, there are patients who need to start ART quickly because they are in an advanced stage of infection and have low CD4 cell counts. They start with primary PCP prophylaxis and, if they tolerate this well, ART is initiated approximately fourteen days later. The second group of patients comprise those whose HIV-infection has been diagnosed in an earlier stage of the disease and who have been monitored for some time in the clinic. Once patients approach the CD4 criteria to start ART, these patients are informed that it is time to initiate treatment and they are usually given (at least) a few weeks to prepare themselves.

The adapted start-up phase of the intervention for treatment initiating patients consists of the following: During the preparatory visit approximately two weeks prior to ART initiation, the patient and nurse go through steps 1–4 of the AIMS-protocol. Patients are advised that finding a good routine immediately can be challenging, and they are therefore provided with a choice to start with a “*readiness period*”. This readiness period means that patients are given a MEMS-view cap with their prescribed primary PCP prophylaxis (for patients with low CD4 count) or fourteen multivitamins/vitamin C tables (for the other intervention patients) for the next fourteen days, and try to follow the medication intake schedule that they would also use when starting with ART. Patients then return to the clinic, MEMS-data is downloaded and discussed with the HIV-nurse, and problems and successes are discussed. The main objective of this readiness period is to offer patients the opportunity to develop some intake routines prior to initiating ART, to identify and address key barriers, and it allows the patient and health care provider to examine whether the patient is really ready to start. Patients who do not want this practice period commence the MEMS-monitoring directly with ART.

### The control group: mapping usual care

Usual adherence care in the Netherlands is primarily delivered by HIV-nurses. In addition to supporting adherence, HIV-nurses may discuss patients’ sexual behaviours as well as their physical, social, and mental well-being. Hence, adherence counselling is only one of the tasks, although an important one, of HIV-nurse practice.

In published manuscripts of adherence interventions, the usual care provided to control groups is typically described using just two words: ‘usual care’ [12]. However, the active content of usual care may vary considerably between study sites, can influence adherence and consequently clinical outcomes. Variability in the ‘quality’ of usual care has been shown to explain approximately 34% points of the difference in treatment success rates among control groups only [12], and influences the effect sizes obtained by the experimental interventions [13]. Assessing usual care and controlling for variability in usual care in the analyses is therefore an important element of the current study.

In order to determine the content of usual adherence care, all HIV-nurses are asked to fill out a semi-structured questionnaire with nine open questions covering the major categories of adherence support counselling [12]. The questionnaire explores the adherence care that nurses systematically deliver to all of their patients (i.e., nurses’ usual care). The reported activities are then coded using a taxonomy of behaviour change techniques, previously used to reliably code intervention and usual care content in meta-analyses of adherence interventions (the taxonomy is available at http://www.marijndebruin.eu/Meta/HIVadherence/Taxonomy) [12,13]. The questionnaire is completed at baseline (prior to the nurses’ training in the AIMS intervention) and after study completion.

### Measures and flow of treatment-experienced patients

The design and measures are graphically displayed in Figure 1 and in Table 1.

**Table 1:** Overview of measurements per time point separate for treatment-experienced and initiating patients

**Treatment-experienced patients**		**T = −1**	**T = 0**	**T = 1**	**T = 2**	**T = 3**
Demographic characteristics			x			x
Plasma viral load			x	x	x	x
Health care consumption (HCC)			x	x	x	x
Quality of life (SF-12v2)			x	x	x	x
Adherence (MEMS-data)*		x**	x	x	x	x
**Treatment-initiating patients**	**T = 0**	**T = 1**	**T = 2**	**T = 3**	**T = 4**	**T = 5**
Demographic characteristics	x					x
Plasma viral load	x		x	x	x	x
Health care consumption (HCC)	x		x	x	x	x
Quality of life (SF-12v2)	x		x	x	x	x
Adherence (MEMS-data)*	x**	x	x	x	x	x

* MEMS-use varies per treatment arm. For specific details regarding MEMS-use, see ‘measurements and patient flow’ section in text and Figures 1 and Figure 2.

** Start of MEMS-measurement.

T = −1 (recruitment): The HIV-nurses approach eligible patients and inform them verbally and in writing about the content and objectives of the project and tasks per treatment arm. If approached patients refuse to participate, reason(s) are noted. Consenting patients commence with a 2-month baseline MEMS-monitoring period.

T = 0 (randomisation): After the baseline monitoring period, patients with adequate adherence (100% QD, >95% BID) who are not assigned to control group 1 or 2 are shown their MEMS-results, and then excluded from further study procedures. Those with suboptimal adherence are randomised to the intervention group, to control group 1 (with MEMS-monitoring) or control group 2 (without MEMS-monitoring). Patients receive information on the further study procedures and also complete Questionnaire 1: QoL (SF-12v2), Health Care Consumption (HCC), and demographic characteristics. Patients assigned to the intervention group receive a MEMS-cap or a MEMS-view cap (optional) plus the AIMS-intervention, and continue using the MEMS-cap during the next 4–5 months. Patients assigned to control group 1 are asked to use the MEMS-cap at least 2 months prior to the following visit (without seeing the data). Patients assigned to control group 2 hand in the MEMS-cap now and continue receiving usual care.

T = 1: 4–5 months after randomisation (depending on their usual visit interval) patients return for the next visit. The intervention group receives AIMS. All patients fill out Questionnaire 2 (HCC + SF-12v2). Patients assigned to the intervention group are asked to continue using the MEMS-cap at least during the 2 months prior to the following visit (T = 2).

T = 2: 8–10 months later (depending on their usual visit interval) patients return for the next visit. The intervention group receives AIMS. All patients fill out Questionnaire 3 (HCC + SF-12v2). Patients assigned to the intervention group and control group 1 continue using the MEMS-cap at least during the 2 months prior to the following visit (T = 3).

T = 3: The visit at month 12–15 after randomisation is focused on completing trial participation. Patients in the intervention group receive AIMS and are offered the opportunity to continue using the MEMS-(view)cap and AIMS. Patients in control group 1 may decide to continue using the MEMS-(view)cap and are offered to receive AIMS during the next non-study visit. All patients fill out Questionnaire 4 (SF-12v2, HCC, demographic characteristics).

### Measures and flow of treatment-initiating patients

The design and measures are graphically displayed in Figure 2 and in Table 1. There are a few differences compared with the procedures described for the treatment-experienced patients. Patients visit the clinic at inclusion and randomisation (T = 0), at 2–4 weeks (T = 1), 3–4 months (T = 2), 6–7 months (T = 3), 9–10 months (T = 4), and at 12–14 months (T = 5).

T = 0 (recruitment and randomisation): The HIV-nurses approach eligible patients and inform them verbally and in writing about the content and objectives of the project, and the tasks depending on the arm of the assignment. If approached patients refuse to participate, reason(s) are noted. Consenting patients are randomised. Patients complete Questionnaire 1 (SF-12v2, HCC, demographic characteristics) at treatment initiation. Those randomised to the intervention group receive the MEMS-view cap and commence the AIMS start-up protocol, or start directly with ART. Those randomised to control group 1 or 2 follow usual care procedures.

T = 1-T = 5: At T = 2, T = 3, and T = 4, they complete Questionnaire 2, 3, and 4 (HCC + SF-12v2). At T = 5, they receive Questionnaire 5 (SF-12v2, HCC, demographic characteristics). Patients assigned to the intervention group and starting with ART continue using the MEMS-view cap and receive AIMS support up to month 3–4 (T = 2). During the next two periods (up to T = 3 and T = 4) patients assigned to the intervention group continue using the MEMS-cap at least during the last month prior to the following visit. The last 3–4 months of the study (from T = 4 to T = 5) is a follow-up period and intervention patients are required to use the MEMS-cap during at least 2 months prior to the follow-up visit. Patients in control group 1 (controls with MEMS) are asked to use the MEMS-cap during the first 3–4 months of ART (up to T = 2) and during the last two months of the follow-up period (before T = 5). Patients in control group 2 do not use the MEMS-cap.

### Analyses

#### Research question 1) effectiveness evaluation

The proportion of patients with an undetectable viral load (<20, <25, or <40 copies/ml, depending on the clinic’s viral load testing kit) is calculated along with corresponding 95% CIs for three time points (for treatment-experienced patients: T1, T2, and T3; for treatment-initiating patients: T3, T4, and T5) after the baseline for the intervention and control groups, respectively, and plotted as a function of time. Next, a repeated-measures multilevel analysis is conducted with viral load status (undetectable/detectable) at the 3 measurement points as the dependent variable and group membership (intervention/control 1 and 2 together) as the independent variable. The nesting of patients within nurses is accounted for in the model by allowing the (log) odds of an undetectable viral load to vary randomly among nurses. Differences in treatment effectiveness among nurses will be accounted for in the model by inclusion of a corresponding random effect. Depending on the distribution of the outcome, we select the most appropriate model for analyses (i.e. regression analyses with log viral loads, or logistic regression with detectable/undetectable).

For the full sample, the adherence level during the first two months of the intervention and the last two months of the follow-up period as measured with the MEMS-caps is examined in two separate multilevel model including group membership (intervention or control group 1) as the independent variable, and including treatment stage (treatment-experienced or treatment-initiating) as a covariate in the model. We intend to explore whether repeated measures analyses are possible, although this is complicated by the absence of baseline adherence for treatment-initiating patients (so only two repeated measures) and the multi-level design.

#### Research question 2 and 3) trial-based economic evaluation

The objective of the economic evaluation study is to examine whether the delivery of AIMS-intervention compared to care as usual is preferable in terms of costs, effects, and utilities from a societal perspective. The time horizon is the same as the study duration after the AIMS-intervention is initiated (12–15 months).

This economic evaluation involves a combination of a cost-effectiveness analysis (CEA) and a cost-utility analysis (CUA). In a CEA, effects are presented in behavioural (adherence) and clinical (plasma viral load) outcomes. In the CEA, the incremental cost-effectiveness ratio (ICER) is expressed as the incremental costs per percentage of patients with detectable viral load. A brief health care utilisation questionnaire (HCC) is used aimed at identifying all relevant direct and indirect cost aspects (e.g., intervention costs, health care costs, patient costs, and costs outside the health care sector). The valuation of healthcare and patient costs is based on the updated Dutch manual for cost analysis in healthcare research [35]. Costs of medication are calculated using prices based on Daily Defined Dosage (DDD) taken from the Dutch Pharmacotherapeutic Compass. Productivity costs are calculated by means of the friction cost method. Registrations from nurses are used to calculate the time spend on the intervention each consultation. The primary outcome measures for the cost-utility analysis are QALYs, based on the SF-12v2 utility scores [36,37]. In the CUA, the ICER is expressed as the incremental costs per QALY. To demonstrate the robustness (checked by non-parametric bootstrapping) of our base-case findings a multi-way sensitivity analysis is performed (e.g., by varying cost-prices and volumes between minimum and maximum). The bootstrapped cost-effectiveness ratios are plotted in a cost-effectiveness plane. The bootstrapped ICERs are also depicted in a cost-effectiveness acceptability curve showing the probability that the AIMS intervention is cost-effective using a range of ceiling ratios.

#### Research question 4) model-based economic evaluation

The time horizon of the Cost-Utility Analysis (CUA) is extrapolated towards the remaining life expectancy of the study population. The CUA is of major importance since the impact of AIMS on viral load, costs, and QoL reaches beyond the 12-month study period of the RCT. A Markov Monte Carlo decision analytic model is developed to calculate lifetime incremental costs and incremental QALYs of AIMS in comparison with usual care. The model combines the results of the clinical study and data from the medical literature. In the modelling study we also perform probabilistic sensitivity analysis to test parameter uncertainty and to construct cost-effectiveness acceptability curves. Future costs and effects are discounted according to the Dutch guidelines for cost calculations in health care [35].

## Discussion

The theory- and evidence-based Adherence Improving self-Management Strategy (AIMS) is a nurse-based intervention that has been demonstrated to improve adherence and viral suppression rates in a randomised controlled trial. However, evidence on its cost-effectiveness is lacking. Following a recent review suggesting that cost-effectiveness evaluations of adherence interventions for chronic diseases are rare and that their methodology is poorly described in the literature, this manuscript presents the study protocol for the economic evaluation of AIMS.

The present multi-centre RCT is designed to provide sound methodological evidence regarding the effectiveness and cost-effectiveness of AIMS to support treatment adherence among a large and heterogeneous sample of HIV-infected patients in the Netherlands. The objective of the current paper is to describe the trial protocol of the cost-effectiveness evaluation of the AIMS-intervention that reports the information required to fully evaluate the study design on methodological quality. Comprehensiveness of evidence should be optimal to guide implementation decisions efficiently. The AIMS-study is therefore designed to be consistent with the Drummond checklist, Cochrane guidelines, and CONSORT guidelines for trial reporting [29,30,38].

A large part of behaviour change counselling in daily practice is offered by nurses. Despite that they can offer high-quality adherence support, there is rarely high-quality evidence available in support of this important work. We hope that the AIMS-study can contribute to this evidence-base by demonstrating the efficiency of adherence counselling by well-trained nurses. We realise, however, that the practical value will eventually depend on the success of wide-scale implementation and continuation of AIMS. We therefore expect, in case of proven cost-effectiveness, to develop a dissemination project with the relevant stakeholders and to use the results of this study for advancing usual care guidelines.

### Trial status

Active, recruitment recently finalised.

### Consent

Written informed consent was obtained from the patients for the publication of this report and any accompanying images.

## Abbreviations

AIMS: Adherence improving self-management strategy; ART: Antiretroviral therapy; BID: Twice daily dosing; CD4: Cluster of differentiation 4; CUA: Cost-utility analysis; CUE: Cost-effectiveness analysis; DDD: Daily defined dosage; HCC: Health care consumption; HIV: Human immunodeficiency virus; ICER: Incremental cost-rffectiveness ratio; MEMS: Medication event monitoring system; PCP: Pneumocystis carinii pneumonie; QALY: Quality-adjusted life year; QD: Once each day dosing; QoL: Quality of life; RCT: Randomised controlled trial; SF-12v2: Short from health survey (12 items, second version).

## Competing interests

The authors declare that they have no competing interests.

## Authors’ contributions

The conception of idea for the AIMS-study was from authors MdB, HS, and JP. The study was designed by three behavioural scientists (authors MdB, JM, and HS), an infectiologist (author JP), a nurse practitioner (author HEN), two health economists (authors SE and EO), and three statisticians (authors WV, LG, and ET). All authors read, edited and approved the final manuscript.

## Pre-publication history

The pre-publication history for this paper can be accessed here:

http://www.biomedcentral.com/1472-6963/13/274/prepub
